# Van der Waals Engineering of One-Transistor-One-Ferroelectric-Memristor Architecture for an Energy-Efficient Neuromorphic Array

**DOI:** 10.1021/acs.nanolett.4c06118

**Published:** 2025-02-03

**Authors:** Yinchang Ma, Maolin Chen, Fernando Aguirre, Yuan Yan, Sebastian Pazos, Chen Liu, Heng Wang, Tao Yang, Baoyu Wang, Cheng Gong, Kai Liu, Jefferson Zhe Liu, Mario Lanza, Fei Xue, Xixiang Zhang

**Affiliations:** † Physical Science and Engineering Division, King Abdullah University of Science and Technology, Thuwal 23955-6900, Saudi Arabia; ‡ Department of Mechanical Engineering, The University of Melbourne, Parkville, VIC 3010, Australia; § Department of Electrical and Computer Engineering and Quantum Technology Center, University of Maryland, College Park, Maryland 20742, United States; ∥ Physics Department, Georgetown University, Washington, D.C. 20057, United States; ⊥ Department of Materials Science and Engineering, National University of Singapore, Singapore 117575, Singapore; # Center for Quantum Matter, School of Physics, Zhejiang University, Hangzhou 311215, China; 7 ZJU-Hangzhou Global Scientific and Technological Innovation Center, Zhejiang University, Hangzhou 311215, China; 8 Intrinsic Semiconductor Technologies, Ltd., Buckinghamshire HP18 9SU, United Kingdom; 9 Singapore Institute for Functional Intelligent Materials, National University of Singapore, Singapore 117544, Singapore; 10 Electrical and Computer Engineering, King Abdullah University of Science and Technology, Thuwal 23955-6900, Saudi Arabia

**Keywords:** 2D ferroelectric crystals, one-transistor-one-memristor, van der Waals assembling, gate-tunable synaptic behaviors

## Abstract

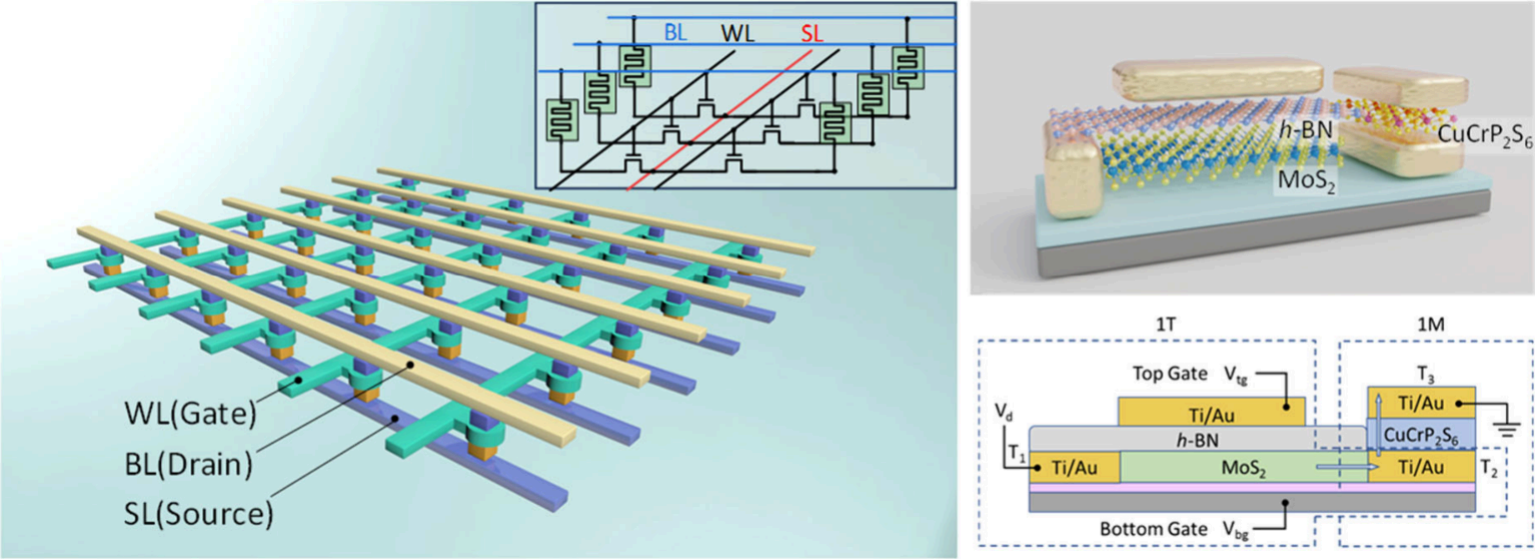

Two-dimensional-material-based memristor arrays hold promise for data-centric applications such as artificial intelligence and big data. However, accessing individual memristor cells and effectively controlling sneak current paths remain challenging. Here, we propose a van der Waals engineering approach to create one-transistor-one-memristor (1T1M) cells by assembling the emerging two-dimensional ferroelectric CuCrP_2_S_6_ with MoS_2_ and *h*-BN. The memory cell exhibits high resistance tunability (10^6^), low sneak current (120 fA), and low static power (12 fW). A neuromorphic array with greatly reduced crosstalk is experimentally demonstrated. The nonvolatile resistance switching is driven by electric-field-induced ferroelectric polarization reversal. This van der Waals engineering approach offers a universal solution for creating compact and energy-efficient 2D in-memory computation systems for next-generation artificial neural networks.

In the current era of big data, the emergence of artificial intelligence and machine learning demands high-throughput data storage and processing. Memristors have emerged as promising candidates for high-density integrated memory and neuromorphic computing.^1^ Recent advancements have enabled artificial neural networks (ANNs) with synapse-like memristor cells, achieving exceptional performance in data storage and computation for data-centric applications.^2−4^ Among these technologies, two-dimensional (2D) material-based memristors are gaining attention in the semiconductor industry for their scalability, low power operation, and compatibility with existing technologies.^5^ Two-dimensional ferroelectric (FE) memory devices are particularly promising.^6^ These devices exploit polarization and metal/semiconductor interface effects for nonvolatile resistance switching^7^ and offer ultrafast switching time and ultralow power consumption,^8,9^ making them ideal for parallel sensing–storing–computing applications.^10−13^ However, the exploration of ferroelectric-driven device arrays for executing high-volume tasks remains limited.

Building neural networks with memristor arrays faces two major challenges. The first is the leakage current: when the memristor is configured to an “OFF” state, leakage currents still exist due to material imperfections or defects, causing additional static power consumption and impacting the stability of the stored data.^14−17^ The second is the sneak current path: when reading or writing a specific memristor, the current may pass through other parallel cells, forming a “sneaky” pathway and resulting in considerable read–write errors.^16,18^ Fortunately, combining memristors with field-effect transistors can help alleviate these issues,^19^ thereby avoiding crosstalk problems and improving computational efficiency.^20−23^ Yet, the hybridization of memristors and transistors via all-2D assembly is scarcely studied.

Herein, we demonstrate a novel one-transistor-one-ferroelectric-memristor (1T1M) device^24^ by assembling CuCrP_2_S_6_, hexagonal boron nitride (*h*-BN), and MoS_2_. Different from previous studies,^25,26^ our 1T1M device exhibits gate-tunable nonvolatile resistance switching due to electric-field-driven FE transition in CuCrP_2_S_6_.^13,27,28^ The 1T1M architecture is entirely composed of 2D van der Waals (vdW) crystals, leveraging their atomic-scale thickness and sharp interfaces. The active memristive material CuCrP_2_S_6_ belongs to the emerging family of transition-metal phosphosulfides but has not received considerable attention. The vdW-engineered CuCrP_2_S_6_-based 1T1M exhibits ultralow sneak current (∼120 fA), colossal tunability (10^6^) of resistance states, and low operation voltages (<1 V). Our 1T1M memory array suppresses crosstalk by 2 orders of magnitude in experiments and achieves 90% image-recognition accuracy with low energy consumption (12.77 μW) in a simulated 256 × 10 ANN. This study highlights the potential of 2D FE materials for advanced memory architectures.

As discovered by recent studies,^27,29^ CuCrP_2_S_6_ crystals, despite being antiferroelectric in bulk, exhibit polarization in thin layers under external electric fields through ferroelectric phase transitions. In its structure (Figure 1a), Cu^I^ atoms are fixed on one side of the monolayer, while Cu^II^ atoms shift vertically when sufficient energy is acquired from external stimuli, such as vertical electric fields. This shift enables different phases with distinct symmetries. When Cu^I^ and Cu^II^ are located on the same side, the crystal produces upward or downward polarization at the macroscopic level, i.e., an FE state. Conversely, when Cu^I^ and Cu^II^ appear on opposite sides, their dipole moments cancel each other out, resulting in a state with no macroscopic net polarization, i.e., an antiferroelectric (AFE) state. Band structure calculations confirm monolayer CuCrP_2_S_6_ as a direct bandgap semiconductor with 1.04 and 1.18 eV for AFE and FE states, respectively (Figure S1). Asymmetric electrostatic potential distribution along the *c*-axis (Figure 1b) with Φ_0_ = 3.15 eV provides evidence of the polarization.

**Figure 1:**
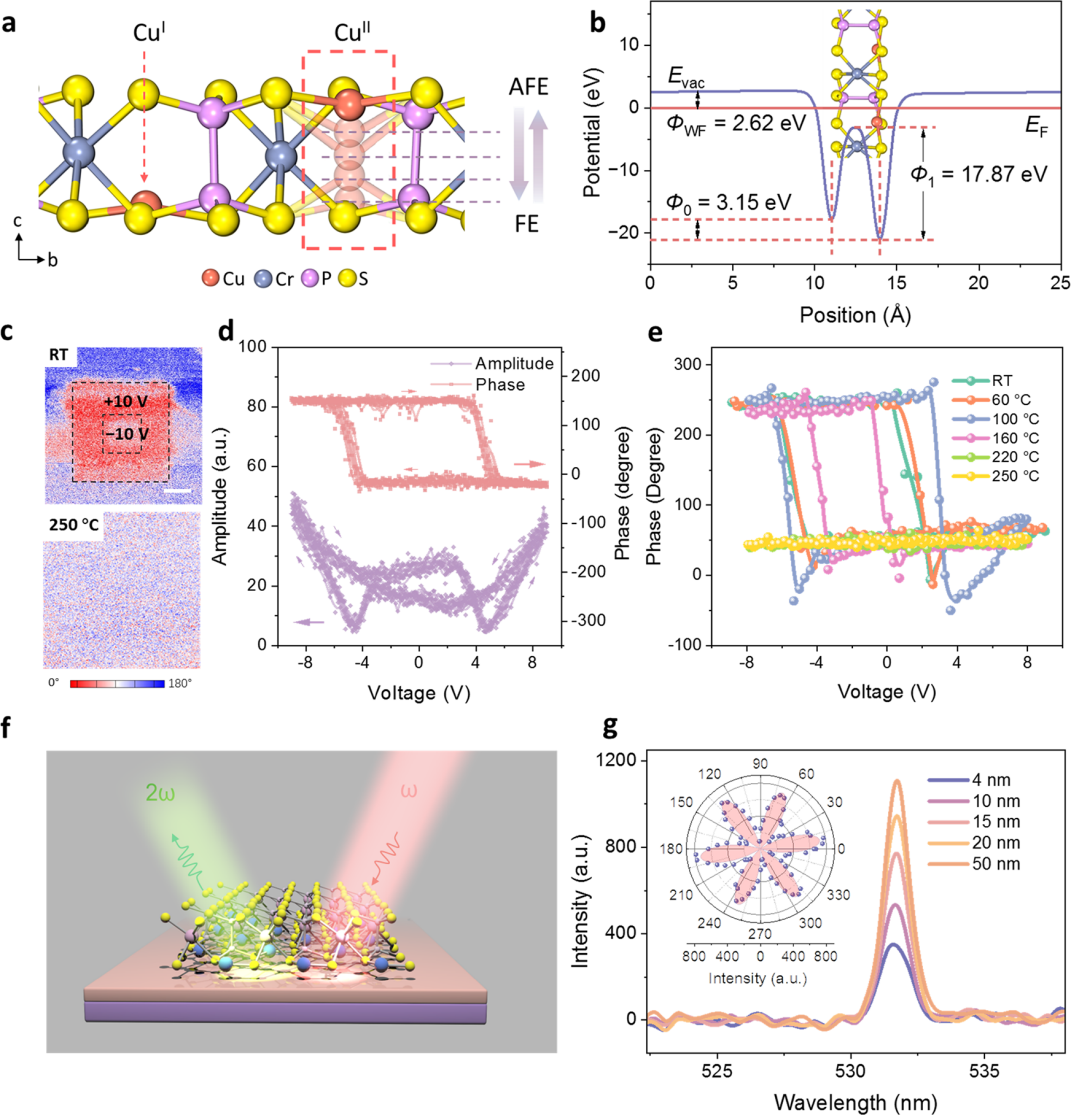
Characterization of CuCrP_2_S_6_ polar properties. (**a**) Crystal structures of CuCrP_2_S_6_ in FE, AFE, and intermediate states indicated by the locations of Cu^II^ atoms along the *c*-axis. (**b**) Electrostatic potential distribution along the *c*-axis of FE CuCrP_2_S_6_ overlaid with its crystal structure. (**c**) PFM-patterned phase images at room temperature and 250 °C. (**d**) Ten continuous cycles of PFM butterfly hysteresis loops observed at room temperature. (**e**) Temperature-dependent phase hysteresis loops. The phase switching vanishes at temperatures of 220 °C or higher. (**f**) Schematic of optical SHG measurements. (**g**) Thickness dependence of SHG intensity. Inset: Polar plot of the SHG intensity when the polarization of the linearly polarized laser is swept from 0 to 360°.

High-quality plate-like CuCrP_2_S_6_ crystals were synthesized using the chemical vapor transport method. Scanning electron microscopy shows plate-like crystals with clear 120° edges (Figure S2a and b), consistent with their hexagonal structure. Energy-dispersive X-ray spectroscopy confirms the uniform distribution of Cu, Cr, P, and S elements (Figure S2c, top) and validates the stoichiometric composition (Figure S2c, bottom). Raman spectroscopy (Figure S2d, top) identifies phonon modes: peak A at 204 cm^–1^ (anion rotation), peak B at 266 cm^–1^ (anion translation), and peaks C and D (stretching of [P_2_S_6_]^4–^), aligning with previous reports.^30^

Electric field-induced polarization behaviors were probed using piezoresponse force microscopy (PFM). The box-in-box pattern was written by applying ±10 V DC biases along the out-of-plane direction, showing clear domain reversal (Figure 1c, top). The phase–voltage and amplitude–voltage curves show hysteresis loops and typical “butterfly” shapes, respectively, indicating the existence of out-of-plane polarization (Figure 1d). The multidomain structure disappears at 250 °C (Figure 1c, bottom), and the hysteresis of phase loops is pronounced with 180° reversal even up to 160 °C but vanishes at 220 °C and above (Figure 1e), both of which indicate polar-to-nonpolar phase transitions.

Second harmonic generation (SHG) probes the non-centrosymmetry, a prerequisite of polarization.^31^ SHG has advantages over PFM in the aspect of being noncontact, nondestructive, and highly efficient. We utilized SHG measurements as an efficient method to screen CuCrP_2_S_6_ flakes across the whole wafer. SHG emission (Figure 1f and g) exhibits a six-petal polar plot, consistent with hexagonal crystal symmetry. The SHG signal increases with the increasing thickness of the sample (Figure 1g), indicating SHG is a bulk property arising from the inherent non-centrosymmetry of the crystal, rather than a surface-induced symmetry breaking phenomenon.

Seamless integration of memory and logic functions in 1T1M configuration requires several considerations to minimize mismatch issues and ensure collaborative operations:^32^ (1) interference between the transistor and memristor;^33^ (2) matching of the working current range and operating voltages;^34^ and (3) contact problems.^35^ Failing to meet matching conditions may limit the full expression of the memory effect and programmable functions. For example, filamentary memristors often require large forming current to establish conductive paths,^6^ which many transistors cannot provide; the nonlinear output characteristics caused by the transistor contacts will hinder the realization of linear multilevel resistance states.^35^ Meanwhile, the stray fields, parasitic capacitance, and defect charging in transistors may bring a negative impact to the high-frequency operation of memristors.^36^ Among the vast library of materials, we found that the recently discovered ferroelectric CuCrP_2_S_6_, when integrated with the MoS_2_ transistor, has immense potential in addressing these critical challenges. Its unique properties such as low switching energy barrier,^37^ strong polarization,^28,37−39^ minimal stray field,^29^ and low degradation rate, make it a perfect choice for collaborative operation with MoS_2_ FETs. Furthermore, the multidomain nature of CuCrP_2_S_6_^40^ collaborates well with the linear output and high current tolerance of MoS_2_ FETs, ensuring the comprehensive exploitation of synaptic properties of memristors.

Our design features a planar-transistor-vertical-memristor cascaded layout, enabling the compact and reliable integration of logic and memory functions with reduced interference. Atomic-thick MoS_2_ is used as the transistor channel due to its high mobility for fast switching; *h*-BN with a wide bandgap serves as both top-gate dielectrics and isolation layers. For the 1T1M configuration, the planar transistor component is assembled by vdW stacking of MoS_2_ and *h*-BN, while the CuCrP_2_S_6_ flake in the vertical memristor is assembled on top of the electrode (T_2_) via vdW forces. The device schematics are displayed in Figure 2a and b. The fabricated device is shown in the scanning electron microscopy image (Figure 2d). The thicknesses of MoS_2_, *h*-BN, and CuCrP_2_S_6_ are 10, 20, and 18 nm, respectively (Figure S3a). The metals for the electrode are Ti (10 nm) and Au (60 nm). The left side of the device, marked by dashed lines and noted as “1T”, serves as a transistor. To mitigate the generation of parasitic capacitance, the gate electrode needs to be patterned in a position that does not overlap with the source (T_2_) and drain (T_1_) electrodes. The cross-sectional transmission electron microscope images and energy-dispersive X-ray spectroscopy confirm the layered structure and sharp interfaces (Figures 2c and S4).

**Figure 2:**
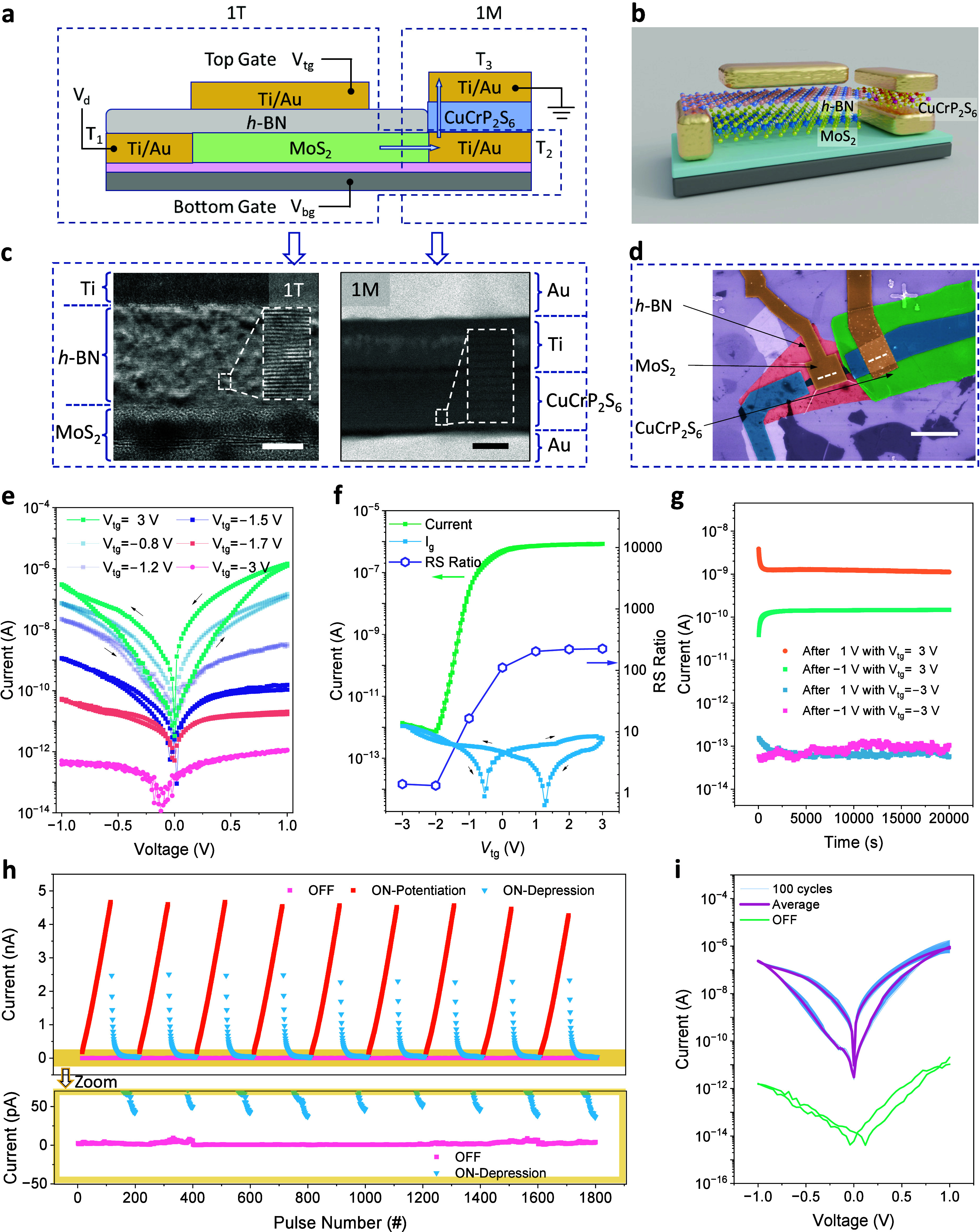
Design of the 1T1M architecture and device characterization. (**a**) Cross-sectional schematic of the 1T1M device structure. The currents were acquired by applying biases onto *V*_d_, and the forward current pathway is marked by arrows. Ti is used for contact. (**b**) 3D schematic illustration of the device structure. (**c**) Cross-sectional transmission electron microscope images of 1T and 1M sections. Scale bar: 10 nm. (**d**) Scanning electron microscope image of the as-fabricated 1T1M device presented in false color. Scale bar: 15 μm. (**e**) Top-gate-tunable memristor: Hysteretic *I*–*V* curves for the 1T1M with *V*_tg_ varied from −3 to 3 V. (**f**) Top-gate transfer characteristics of the 1T1M device. The nonhysteretic transfer curve shows that the “ON” and “OFF” states of the transistor can enable and disable resistance switching, respectively. *V*_d_ = 1 V. (**g**) Retention tests after a pulse of *V*_d_ = ±1 V/10 s with *V*_tg_ set as 3 V and −3 V. The read voltage is 0.1 V. (**h**) Potentiation and depression of the device with *V*_tg_ = 3 V (ON state) and *V*_tg_ = −3 V (OFF state), using pulses of (1 V, 300 ms) for potentiation and (−0.2 V, 300 ms) for depression. Read voltages *V*_d_ = 0.1 V. (**i**) 100-cyle *I*–*V* sweeping test.

Electrical characterization (Figure 2e) shows hysteretic *I*–*V* curves in the “ON” state (*V*_tg_ = 3 V with a floating back gate). The temperature-dependent memristive behavior is illustrated in Figure S5. The positive *V*_tg_ increases the carrier concentration, allowing current flow between T_1_ and T_2_. This causes a significant potential drop across the memristor, generating an electric field strong enough to induce FE polarization reversal. Conversely, when a negative top-gate voltage (*V*_tg_ = −3 V) is applied, the current sharply decreases and the resistance switching window disappears. The negative *V*_tg_ suppresses channel conductivity, leading to a major potential drop across the channel rather than the memristor, which makes the electric field inadequate to induce or reverse the polarization.

Figure 2f depicts the transfer characteristics of the 1T1M cell at *V*_d_ = 1 V, which shows a low subthreshold-swing value of around 125 mV/dec. While a maximum ON/OFF ratio up to 10^6^ at *V*_d_ = 1 V has been achieved by top-gating, the gate leakage current remains below 1 pA. Furthermore, the back-gating has a similar effect to the top-gating (Figure S6). This design effectively integrates the functions of the memristor and transistor, leading to a well-functioning 1T1M cell. The good performance of the 1T1M cell can be ascribed to the following features of the device: (a) the relatively small LRS and HRS currents that can be effectively controlled by gating; (b) being free from the electroforming process, ensuring the current stays within the tolerable threshold of the transistor; (c) good ohmic contacts (Figure S7) ensuring a large potential difference across the memristor to trigger resistance switching.

The 1T1M device exhibits synaptic dynamics, including potentiation and depression. In the “ON” state (with positive *V*_tg_), the device shows a stepwise conductivity increase with excellent linearity due to ferroelectric domain expansion^41,42^ (Figure 2h). Using positive voltage pulses, 100 distinguishable resistance states are achieved. Following the depression process under negative voltage pulses, the current decreases to 20 pA. With a *V*_tg_ of −3 V, the current is further suppressed to 2 pA, regardless of whether the device undergoes a depression or potentiation. Gating not only activates/quenches the synaptic function but also modulates synaptic response linearity (Figure S8).

Figure S9 presents other key synaptic characteristics, including excitatory postsynaptic current (EPSC), paired-pulse facilitation (PPF), and spike-timing-dependent plasticity (STDP). The EPSC increased with higher voltage pulses (Figure S9a). Paired-pulse tests show enhanced current response at shorter pulse intervals (Figures S9b and c). STDP tests demonstrate the correlation between the timing of pre- and postsynaptic spikes and the corresponding synaptic weight change (Figure S9d), resembling the temporal learning rules in biological systems. The stability of the device is examined over 60 cycles of potentiation and depression (Figure S10), demonstrating minimal variations.

The retention tests (Figure 2g) show the resistance states can persist for more than 20,000 s after a pulse of *V*_d_ = ±1 V/10 s. After a fast relaxation process of 1000 s, the current tends to stabilize, exhibiting nonvolatile LRS and HRS. At *V*_tg_ = −3 V, the current is suppressed below 120 fA. This shows *V*_tg_ provides complete ON–OFF switching for the memristor with the gate tunability of 10^4^ for LRS and 10^3^ for HRS. The static power consumption of the standby cell (*P*_static_ = *I*_OFF_ × *V*_Read_, where *I*_OFF_ is leakage current in the “OFF” state, *V*_Read_ is the read voltage^14,17^) is as low as 12 fW, nearly three orders lower than the 1M configuration (300 pW). Cycling *I*–*V* tests demonstrate endurance over 100 consecutive cycles with small cycle-to-cycle variability (Figure 2i). Figure S11 shows stable resistance switching over 3000 cycles.

The band diagrams for understanding the working principles of the 1T1M memory cell are presented in Figure 3. In the initial state (Figure 3a and d), the intrinsic dipoles inside the CuCrP_2_S_6_ exhibit macroscopically antiparallel alignment, i.e., the AFE state. The resistance state of the device is solely dependent on the gate voltages. Figure 3b,c,e,f depict scenarios where the 1T and 1M are working in coordination. With a positive *V*_tg_, the history of applied *V*_d_ determines the polarization direction, modulating the potential barrier and defining the HRS or LRS state. Applying a negative *V*_tg_ lowers the Fermi level, disabling the device and setting it to the “OFF” state. These band diagrams effectively explain the observed experimental results.

**Figure 3:**
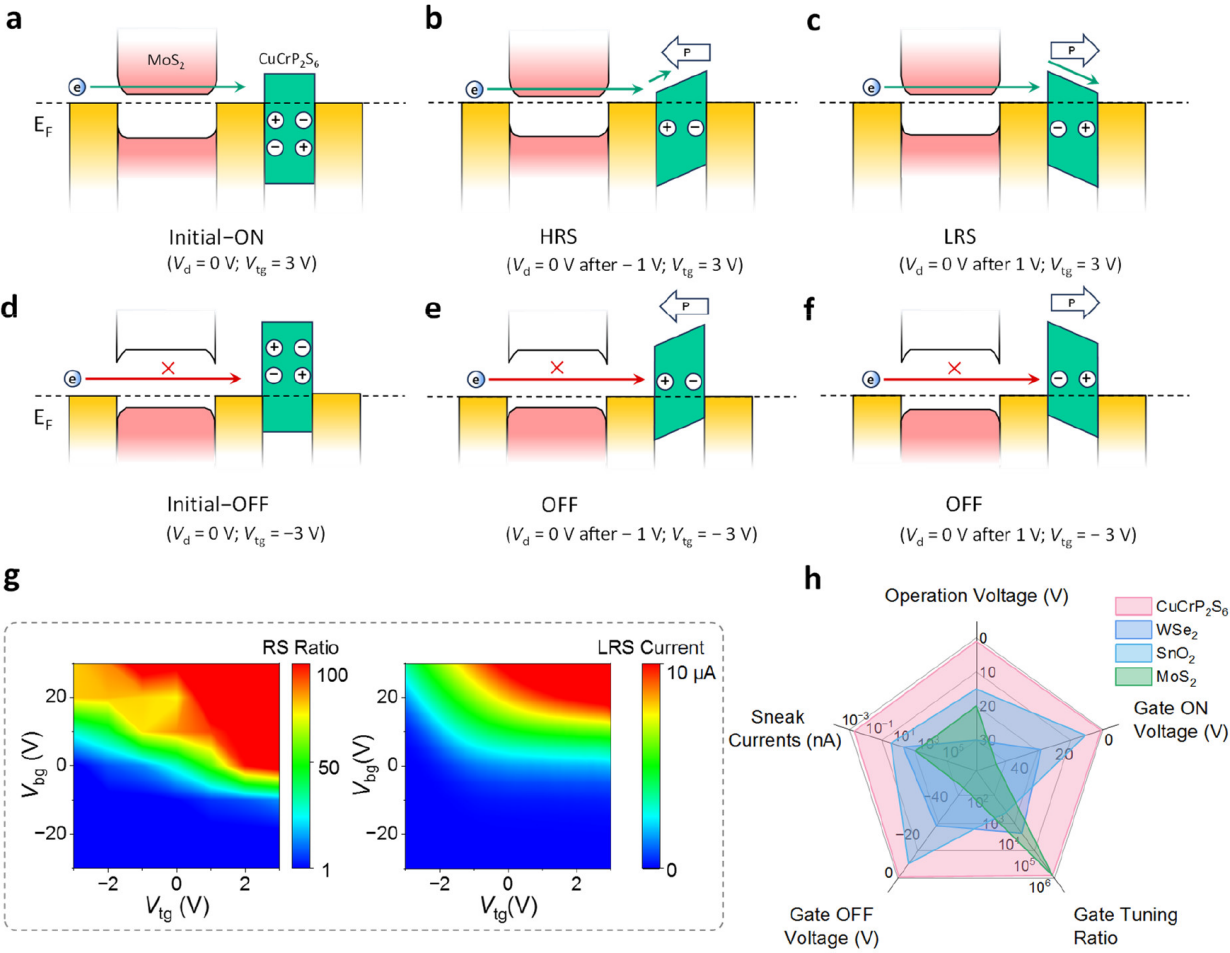
Working principle and performance merits of our design. (**a**–**f**) Energy band diagrams of 1T1M under thermal equilibrium (*V*_d_ = 0 V) with different *V*_tg_-dependent Fermi levels and *V*_d_-dependent polarization directions. (**a**,**d**) and (**b**,**c**,**e**,**f**) depict devices in their initial states and under operation, respectively. “*V*_d_ = 0 V after 1/–1 V” denotes the operations where *V*_d_ is set to 1/–1 V to reverse the polarization, followed by resetting *V*_d_ to 0 V. (**g**) Mapping of top-gate and back-gate voltage dependence of the resistance switching ratio and LRS current. (**h**) Comparison between CuCrP_2_S_6_ 1T1M cells and gate-tunable resistance switching devices in previous reports.

A broader modulation range can be achieved through the collaborative utilization of the back gate and top gate. A positive back-gate voltage (*V*_bg_) combined with manipulations of *V*_tg_ can yield a larger resistance switching ratio (Figure 3g, left). Meanwhile, a negative *V*_bg_ in combination with a varying *V*_tg_ can further suppress the sneak current (Figure 3g, right). With the application of a slightly negative *V*_tg_ (e.g., −1 V), smoother modulations can be achieved by the back-gating. This dual-gating design provides a higher degree of freedom in reconfiguring memristors.

Our 1T1M device potentially addresses the sneak current issue, an unsolved problem in many memtransistor-based designs. While previous reports have achieved gate-tunable resistance switching behaviors in memtransistors,^43−46^ few studies concurrently achieve several desirable features—low-voltage drive, sneak current suppression, and complete quenching of the resistance switching behavior—all within a single unit.^21,47^Figure 3h demonstrates these features in our 1T1M. Compared to multiterminal memtransistors based on vdW materials like WSe_2_,^20^ SnO_2_,^48^ and polycrystalline MoS_2_,^44,49^ our device is operational at smaller gate voltage with significantly reduced sneak current. These substantial improvements mitigate the power consumption challenges for scaled-down applications.

The developed 1T1M architecture is used to construct neuromorphic arrays (Figure 4a). In this design, the bit line (BL) and source line (SL) are connected to the two terminals (T_1_ and T_3_, respectively) of the 1T1M memory cell for read and write operations, whereas the word line (WL) is connected to the gate and is used for targeting specific memristors (Figure 4b). The thicknesses of the materials are listed in Figure S3b. Low cell-to-cell variability is observed in *I*–*V* curves and synaptic features (Figure 4c,d,e). The devices 1–1, 1–2, and 1–3 are made from the same flake, while the devices 2–1, 2–2, and 2–3 are fabricated from another. The 1T1M configuration efficiently eliminates the undesired sneak paths (Figure 4f and 4g) (the in-plane conductivity of CuCrP_2_S_6_ is negligible). *V*_G-OFF_ suppresses conductance in unselected devices (∼1 pS, Figure 4f), significantly reducing sneak current compared to uncontrolled “leaking” states (1 to 100 nS) in arrays without the 1T section (Figure 4g). The sneak current disturbs cell conductance during “writing” operations on adjacent cells, causing crosstalk-induced data loss. Crosstalk impact was analyzed by measuring the cell conductance before and after “write” operations on adjacent cells. For the 1M array (Figure S12), huge conductance discrepancies are observed. In contrast, 1T1M arrays show mitigated fluctuations as the unselected cells are effectively isolated by gating (*V*_G-OFF_ = −3 V). The comparative assessment (Figure 4h) shows conductance variability (defined as Δ*G* = (*G*_1_ – *G*_0_)/*G*_0_, where *G*_0_ and *G*_1_ are conductance before and after crosstalk, respectively) reduced from 800% in 1M arrays to 3% in 1T1M arrays. This demonstrates the important role of the 1T1M design in mitigating crosstalk and enhancing the array performance.

**Figure 4:**
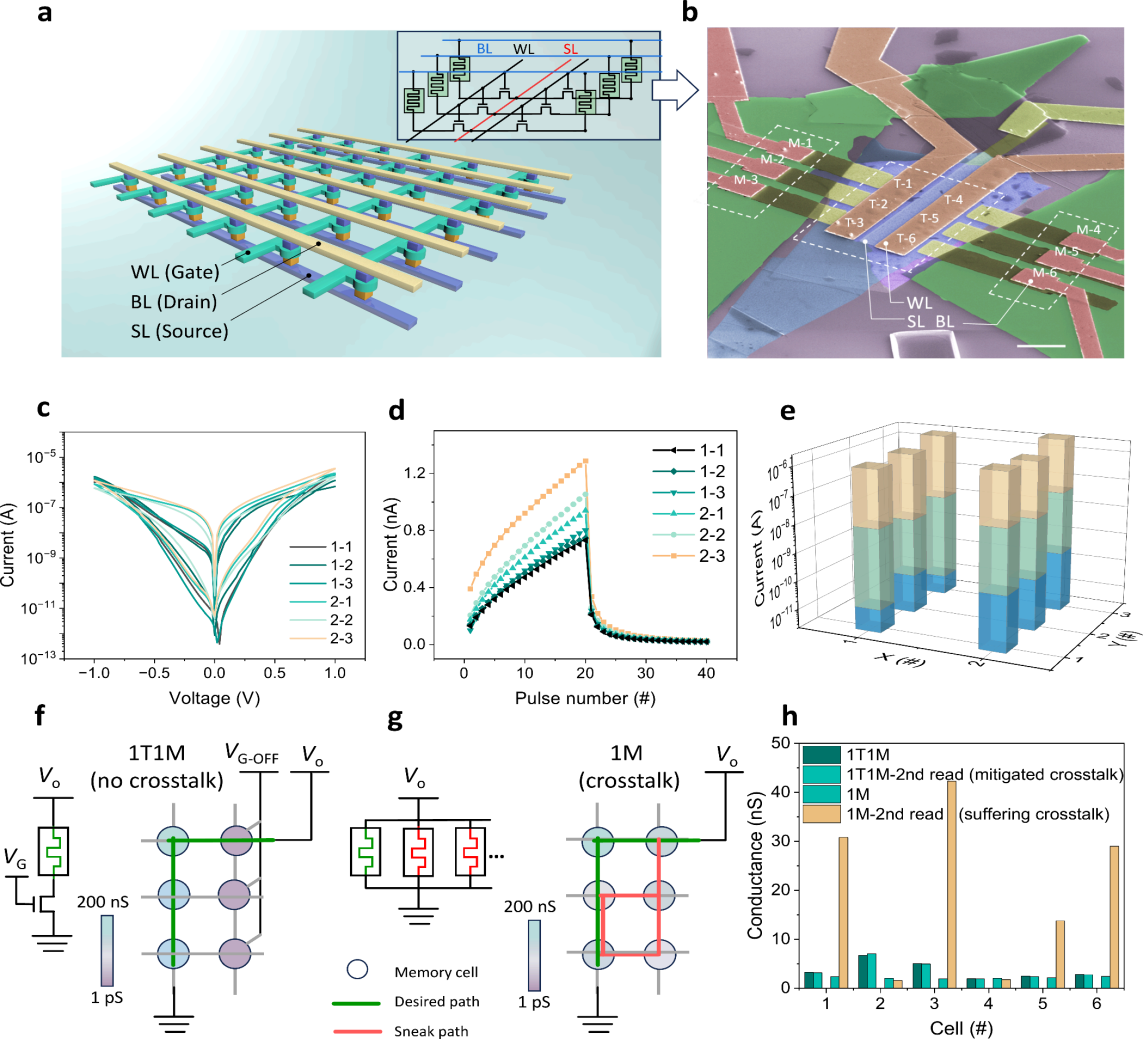
Experimental demonstration of a neuromorphic array with no obvious crosstalk. (**a**) Conceptual schematic of the 1T1M array. Inset: the 2 × 3 miniarray corresponding to the actual fabricated array shown in (**b**). (**b**) False-colored scanning electron microscope image of the miniarray schematically shown in the inset of (**a**). Scale bar: 5 μm. (**c**) Hysteresis *I*–*V* curves for all cells in the 2 × 3 array. (**d**) Potentiation and depression curves for all cells in the array. (**e**) Bar plot of individual cell currents for HRS, LRS (both read at 0.1 V), and maximum value during switching (read at 1 V), extracted from (**c**). (**f**) Conductance of each cell in the 1T1M array when *V*_G-OFF_ = −3 V was applied on the second column. Conductance values (read at 0.1 V) are represented by the color filling the circles (nodes). (**g**) Conductance of cells in a 1M (without 1T) array compared to the 1T1M array in (**f**). The absence of 1T causes undesired sneak paths. (**h**) Comparison of individual cell conductance between the 1T1M array (with reduced crosstalk issues) and the 1M array (suffering crosstalk issues). The conductance measured after the occurrence of crosstalk is noted as “2nd read”.

To justify the advantage of this work, Table S1 compares various types of sneak-controlling 2D devices with different assembly approaches, including one-transistor-four-resistor (1T4R),^50^ one-selector-one-resistor (1S1R),^51^ one-diode-one-selector (1D1S),^52^ and self-selective memtransistor.^49^ These devices are based on different resistance switching mechanisms (charge trapping,^53^ filament,^54^ etc.). These devices demonstrate multiple strategies for cell selection in an array. Unlike others, our work uniquely integrates ferroelectric mechanisms with all-vdW engineering. While semiconductor transistors are known to have low off-state current,^55,56^ the collaborative operation of vdW-engineered transistors and memristors remains a promising area for achieving scalable memory systems. Figure S13 shows current progress toward lower gate voltages and reduced sneak currents. Future research will focus on the scalable material growth and its monolithic integration.^57,58^

Circuit-level simulations were performed with SPICE simulators for CuCrP_2_S_6_ 1T1M crossbar arrays based on experimentally measured *I*–*V* characteristics and weight updates. The arrays came in three different sizes (64 × 10, 128 × 10, and 256 × 10) and were implemented in single-layer perceptron (SLP) neural networks to perform handwritten digit recognition using the MNIST training data set (Figure 5a). The recognition accuracy remains high (∼90%) across all array sizes when the line resistance varies between 1 and 100 Ω (Figure 5b). There is a trade-off between pattern recognition accuracy and energy efficiency. Power consumption decreases dramatically as the line resistance exceeds 1 Ω (Figure 5c) due to its effect on the total current. By optimizing the layout of 1T1M memory units in large-scale arrays, power efficiency can be improved without major accuracy loss. Based on 64 × 10 arrays and 1 Ω resistance, the recognition performance for each input class is shown in the confusion matrix (Figure 5d).

**Figure 5:**
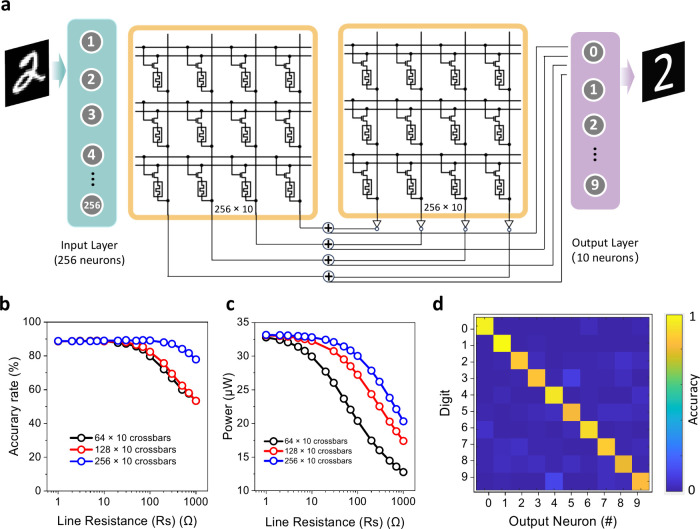
Construction of artificial neural networks. (**a**) Simplified equivalent circuit schematic for 1T1M array-based neural networks. The array is partitioned into left and right sectors, assigned to process positive and negative synaptic weights, respectively. (**b**) Simulated accuracy rate as a function of line resistance for different sizes of neural networks. (**c**) Simulated power consumption as a function of line resistance. (**d**) Confusion matrix of MNIST recognition performance. The rows in the matrix denote the labels of input patterns, the columns represent the output recognition results, and the depth of color represents the recognition accuracy.

In summary, we used vdW engineering to develop a novel all-2D-material-based 1T1M architecture using CuCrP_2_S_6_ ferroelectric polarization, achieving high resistance tunability (10^6^), ultralow minimum sneak current (120 fA), and an ultralow static power (12 fW). This work presents significant progress in all-2D 1T1M arrays with precise cell access and over 2 orders of magnitude suppression in conductance variations. This 1T1M configuration offers universal strategies for energy-efficient and precision-oriented neural networks.

## Data Availability

All data are presented in the main text or Supporting Information.

## Supporting Information Available

Calculated band structures; scanning electron microscope images and energy-dispersive X-ray spectroscopy mapping images; temperature dependence of resistance switching; gate-tunable synaptic behavior tests; EPSC, PPD/PPF, and STDP tests; endurance test; comparison with various multiterminal memory devices; experimental methods (PDF)
