# Supplementary material for: Van der Waals Engineering of One-Transistor-One-Ferroelectric-Memristor Architecture for an Energy-Efficient Neuromorphic Array
Source: Nano Lett. 2025 Feb 3;25(6):2528–37. doi: 10.1021/acs.nanolett.4c06118 (PMC11827105; doi:10.1021/acs.nanolett.4c06118)
Supplement: Supplementary file 1 — nl4c06118_si_001.pdf [file nl4c06118_si_001.pdf]

Supporting Information

Van der Waals engineering of one-transistor-one-ferroelectric-memristor architecture for energy-efficient neuromorphic array

Yinchang Ma[1], Maolin Chen[1], Fernando Aguirre[1], Yuan Yan[2], Sebastian Pazos[1], Chen Liu[1], Heng Wang[1], Tao Yang[1], Baoyu Wang[7], Cheng Gong[3], Kai Liu[4], Jefferson Zhe Liu[2], Mario Lanza[5], Fei Xue[6,7]*, and Xixiang Zhang[1]*

[1]Physical Science and Engineering Division, King Abdullah University of Science and Technology, Thuwal 23955-6900, Saudi Arabia

[2]Department of Mechanical Engineering, The University of Melbourne, Parkville, VIC, 3010, Australia

[3]Department of Electrical and Computer Engineering and Quantum Technology Center, University of Maryland, College Park, MD 20742, USA

[4]Physics Department, Georgetown University, Washington, DC 20057, USA

[5]Department of Materials Science and Engineering, National University of Singapore, Singapore 117575, Singapore

[6]Center for Quantum Matter, School of Physics, Zhejiang University, Hangzhou 311215, China.

[7]ZJU-Hangzhou Global Scientific and Technological Innovation Center, Zhejiang University, Hangzhou, 311215, China.

*Email: xuef@zju.edu.cn; xixiang.zhang@kaust.edu.sa

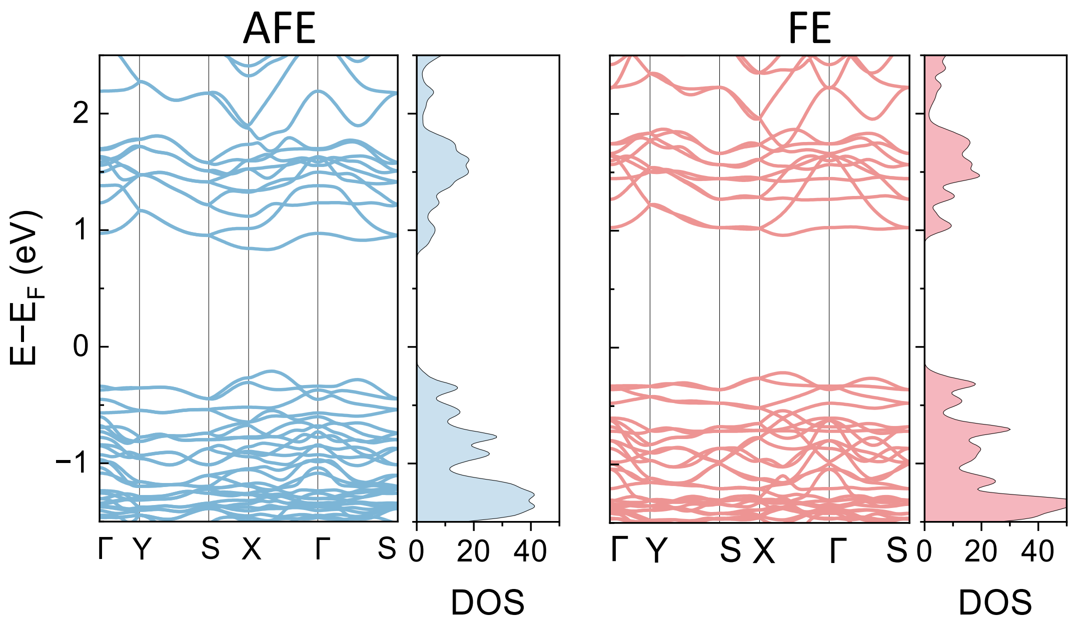

Figure S1. Calculated band structures and DOS for $CuCrP_2S_6$ in both AFE and FE states.

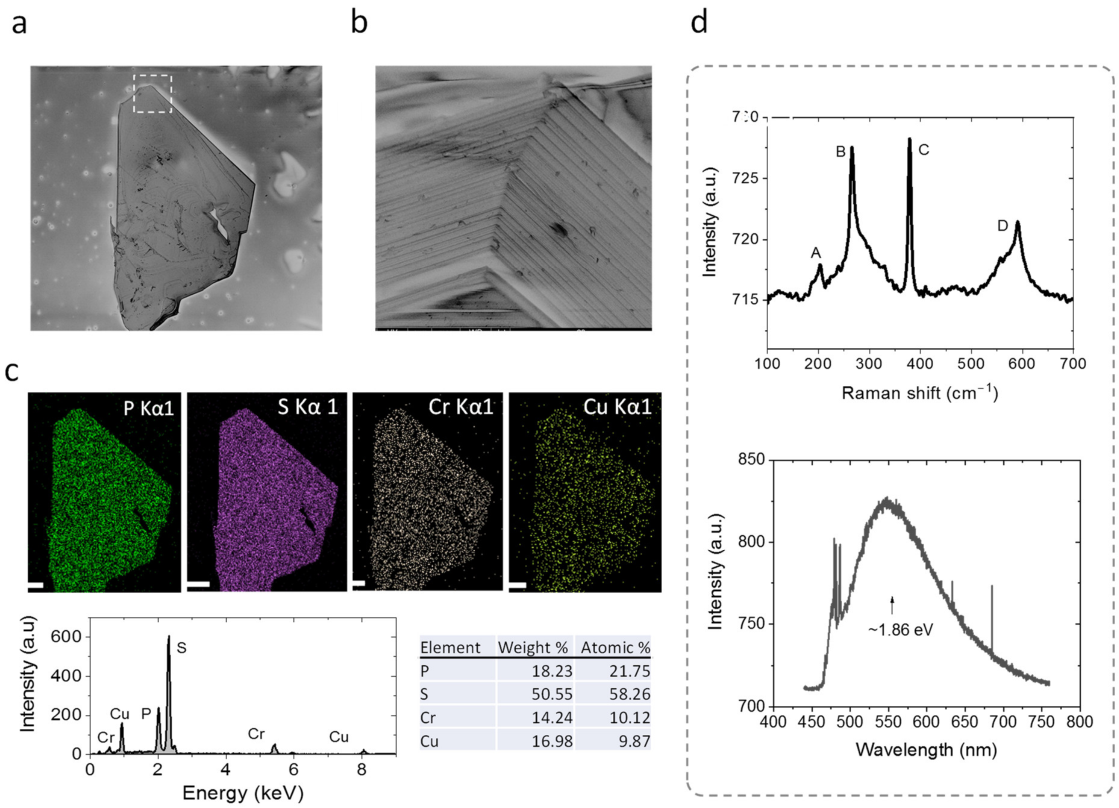

Figure S2. Scanning electron microscope images and energy-dispersive X-ray spectroscopy mapping images of $CuCrP_2S_6$. (**a,b**) scanning electron microscope image of a flake. The area marked by the white dash line in (**a**) is zoomed-in in (**b**); (**c**) Top: energy-dispersive X-ray spectroscopy mapping of P, S, Cr, and Cu elements on the scan flake shown in (**a**). Bottom: energy-dispersive X-ray spectroscopy spectrum and element ratios. (**d**) Raman spectrum (top) and PL spectrum (bottom).

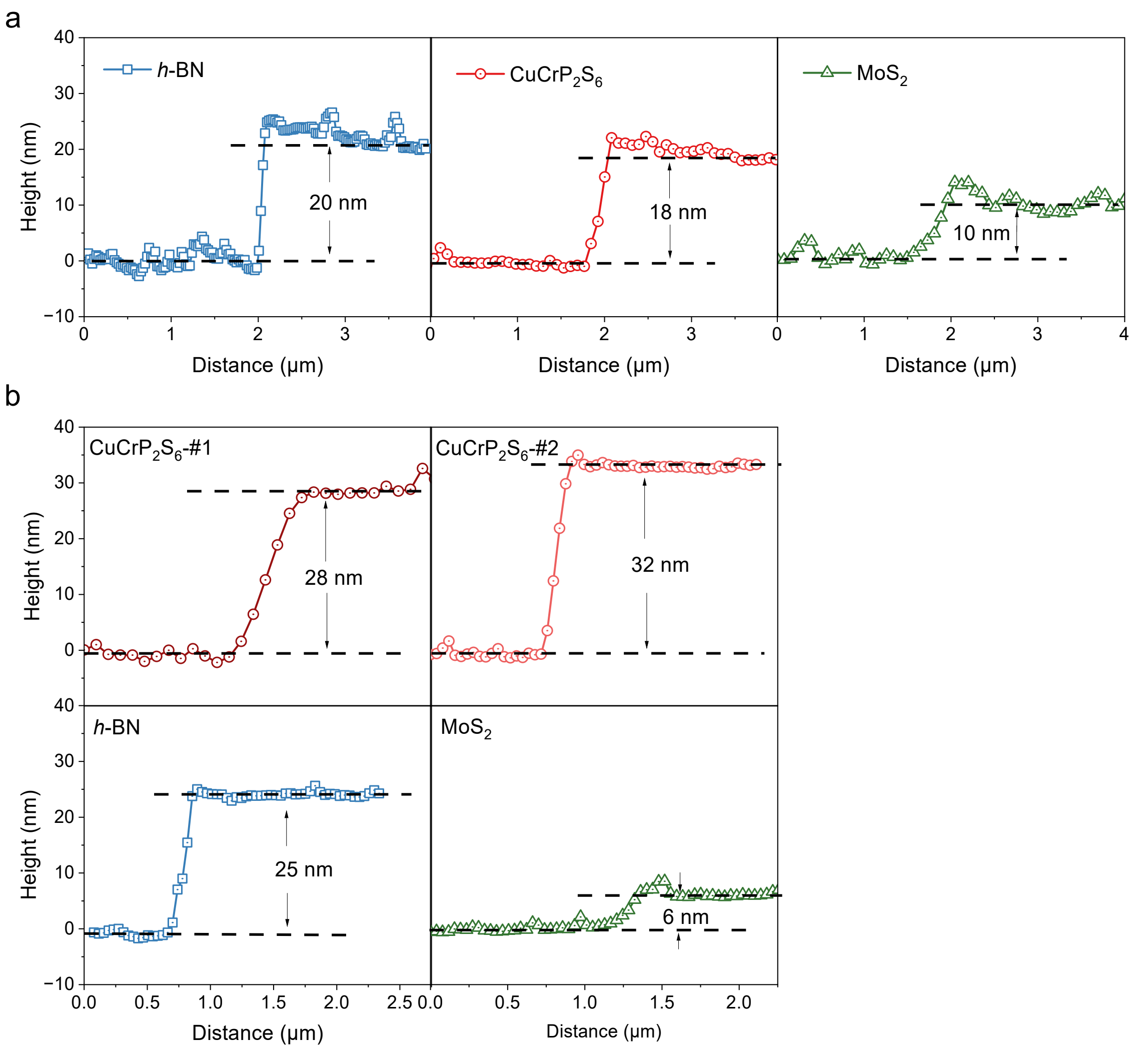

Figure S3. The thicknesses of the materials used to construct the devices presented in Figure 2d and 4b measured by atomic force microscope. $CuCrP_2S_6$ #1 is used for devices 1-1, 1-2, and 1-3; $CuCrP_2S_6$ #2 is used for devices 2-1, 2-2, and 2-3.

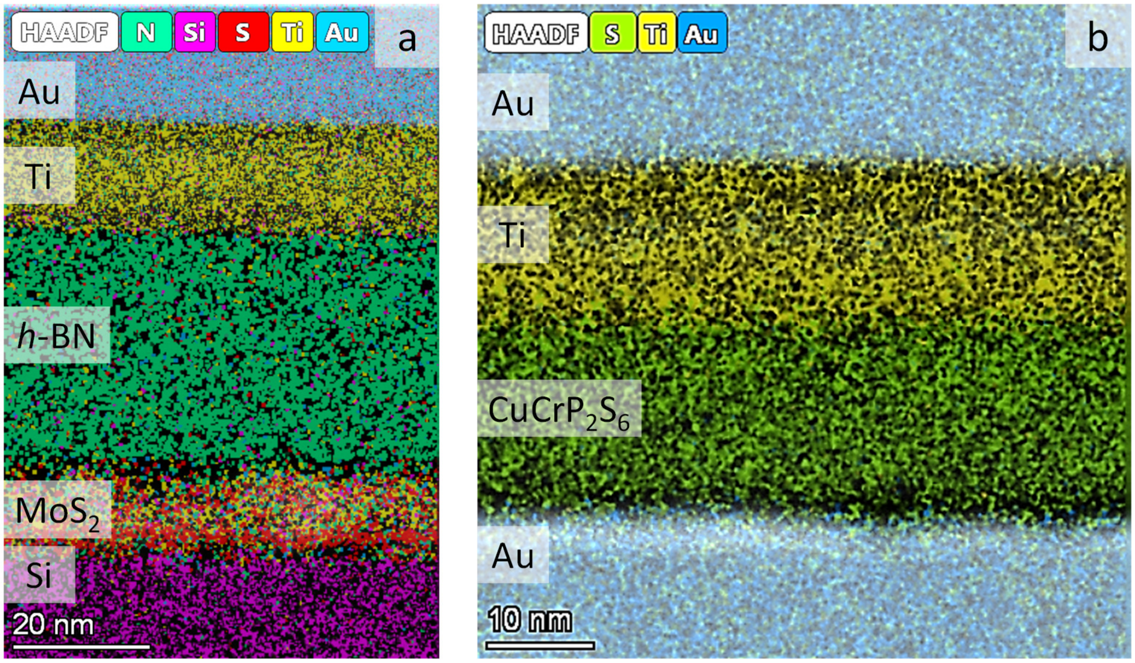

Figure S4. Energy-dispersive X-ray spectroscopy mapping of the cross-section of "1T" and "1M". (**a**) Elemental mapping of N, Si, S, Ti, Au on "1T" section. (**b**) Elemental mapping of S, Ti, Au on "1M" section.

Supplementary Note 1. Temperature dependence of resistance switching

We conducted temperature-variable measurements and found an interesting trend (Figure S5). We divide the temperature range into three parts (Figure S5d) and discuss below.

i. [25℃ ~ 100℃]

We observed the persistence of the switching window up to 100℃ (Figure S5a), agreeing with the persistence of the hysteresis loops. Noticeably, its current increased with increasing temperature, perhaps due to the thermal emission of electrons.

ii. [130℃ ~ 180℃]

The switching window (Figure S5b) shrinks compared with that appears in sub-100℃ region. This is the characteristic reported for ferroelectric memristors. The reason is that thermal energy activates the atoms and enables them to dump between two potential wells, rather than bond into one of them. Meanwhile, the switching window becomes more asymmetric, indicating that the rectifying effect plays a role with increasingly asymmetric interfacial barrier. Especially, the switching window almost vanishes when the temperature approaches 180℃. It vanishes as the temperature increases.

iii. [220℃ ~ 250℃]

The closed switching window reopens after the temperature increases to 220 ℃ and above. Nevertheless, the shape of these I–V curves in this temperature range is largely different from that observed below 100℃, suggesting that a different mechanism, probably ionic migration, starts to play a role. The reopening of the switching window only happens in some devices, while it is absent in the rest of devices. We show both switching case and non-switching case of different devices as a contrast in Figure S5c. That is very possible because Cu atoms are active at high temperatures.

Notably, our memristor characteristics can persist ferroelectric-like resistance switching at a higher temperature (100 ℃) compared with recent popular ferroelectrics $CuInP_2S_6$, which loses its memory effect at 40 ℃. Our close investigation into temperature dependence reveals potential phase transition and further provides insights into the memory characteristics of our device.

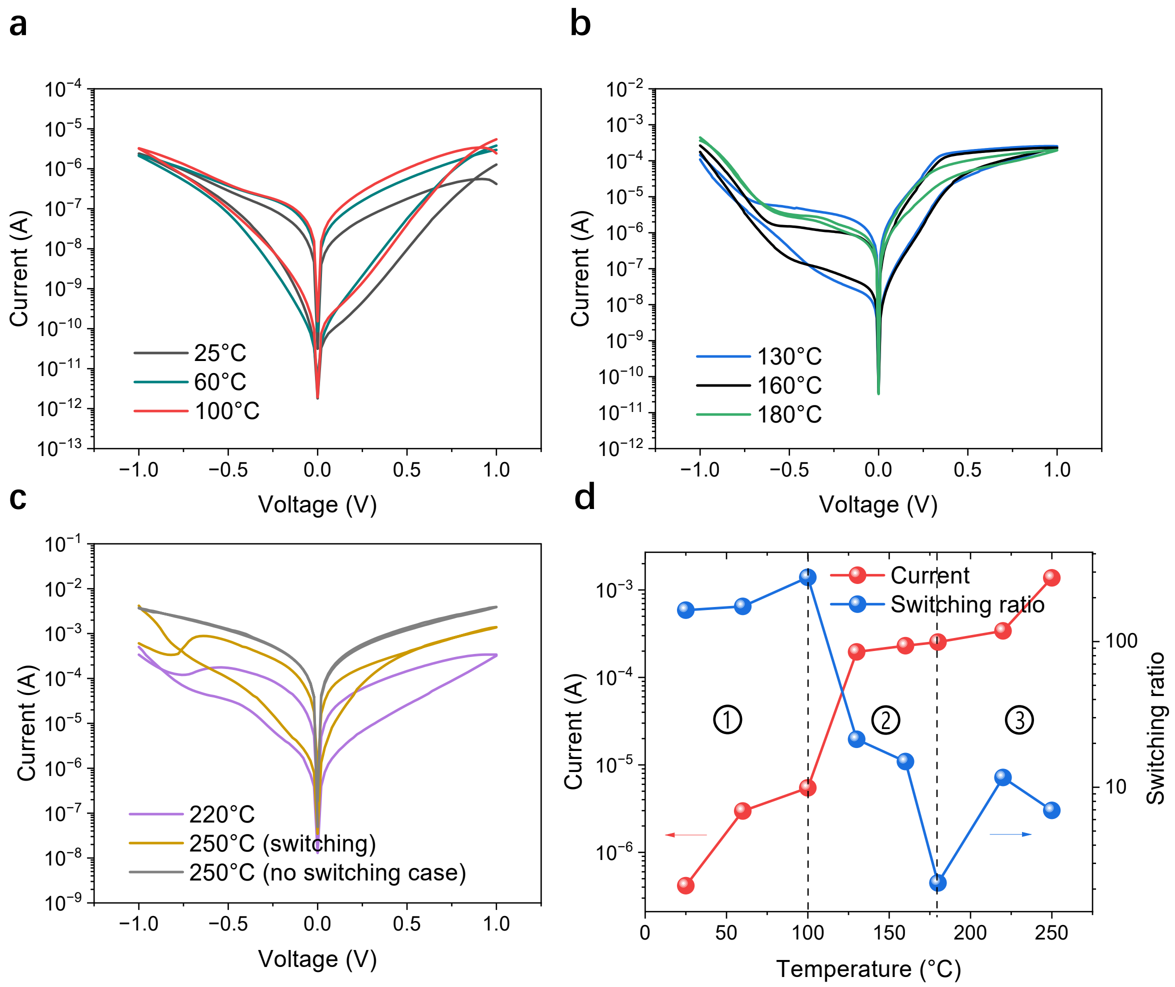

Figure S5. Memory characteristics of $CuCrP_2S_6$ memristor at various temperatures. (**a,b,c**) *I–V* curves in the temperature range from 25 ℃ to 250 ℃. All hysteresis curves are anticlockwise. (**d**) Temperature dependence of current at 1 V and switching ratio.

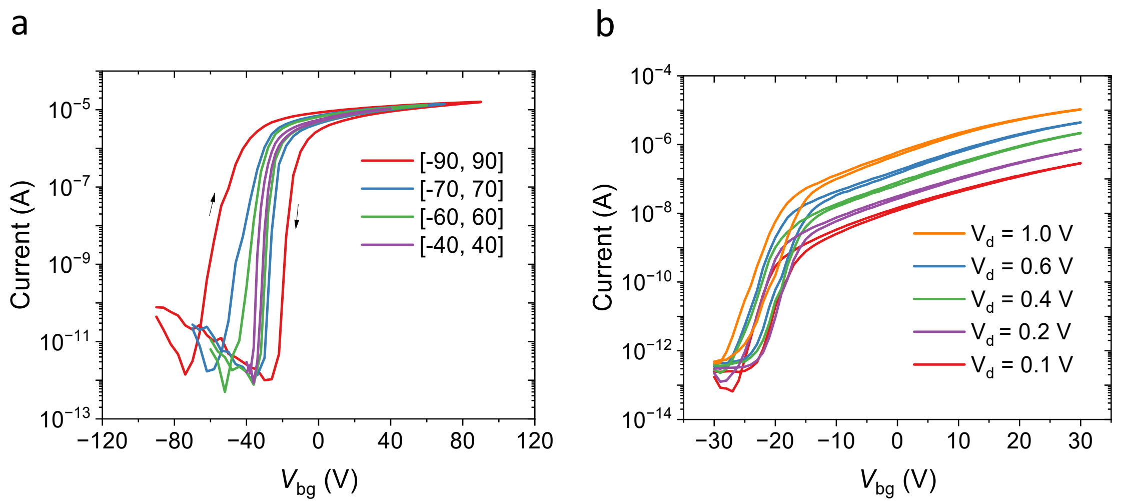

Figure S6. Back-gate transfer characteristics of the 1T1M. (**a**) Transfer curves with different sweep ranges of V_{bg} measured at V_d = 1 V. The clockwise hysteresis may result from charge trapping on the SiO_2 surface. (**b**) Transfer curves with different V_d with V_{bg} sweeping between −30 to 30 V.

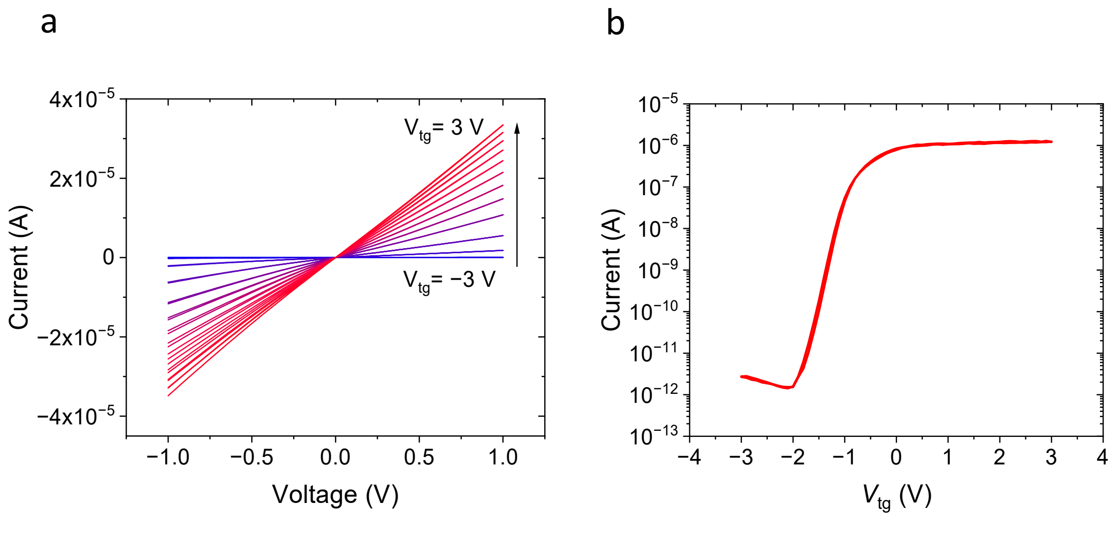

Figure S7. Top-gate output and transfer characteristics of the transistor (“1T”) portion in 1T1M. (**a**) Output curves with increasing V_{tg} from −3 to 3 V with 0.2 V steps. (**b**) Transfer curve of “1T” measured at V_d = 0.1 V.

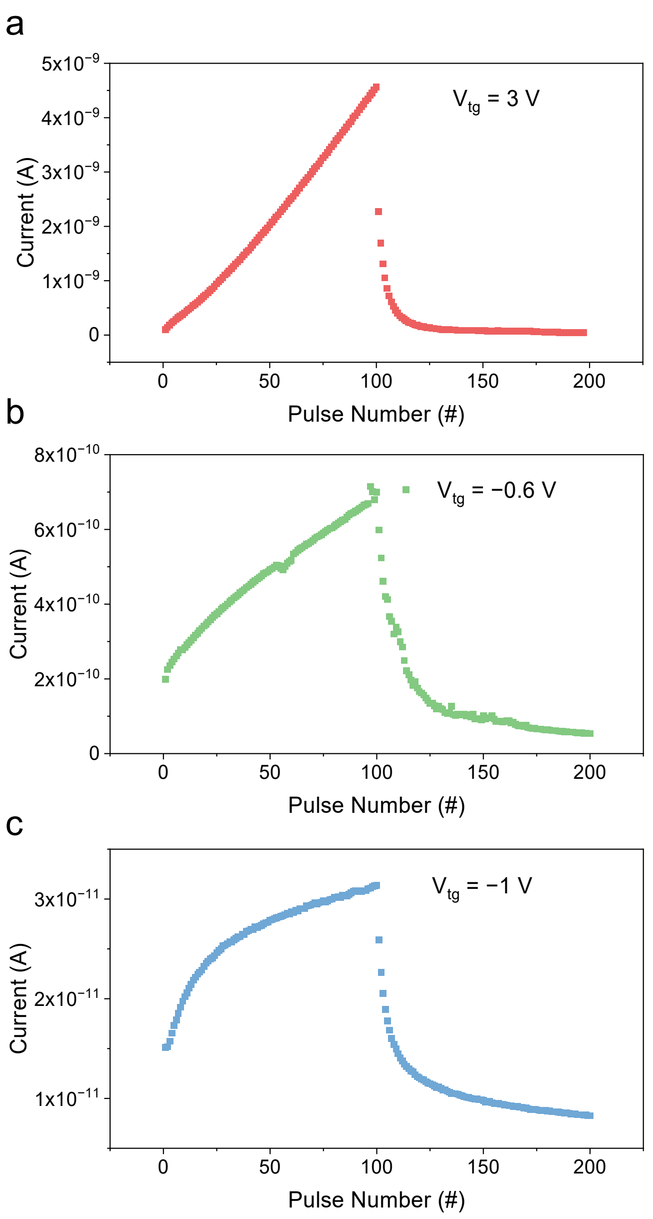

Figure S8. Reconfigurable synaptic behaviors of 1T1M. V_{tg} = 3 V, −0.6 V and −1 V for (**a,b,c**), respectively. Voltage pulses for potentiation and depression are set to be 1 V/300 ms and −0.2 V/300 ms, respectively. Read voltages V_d = 0.1 V.

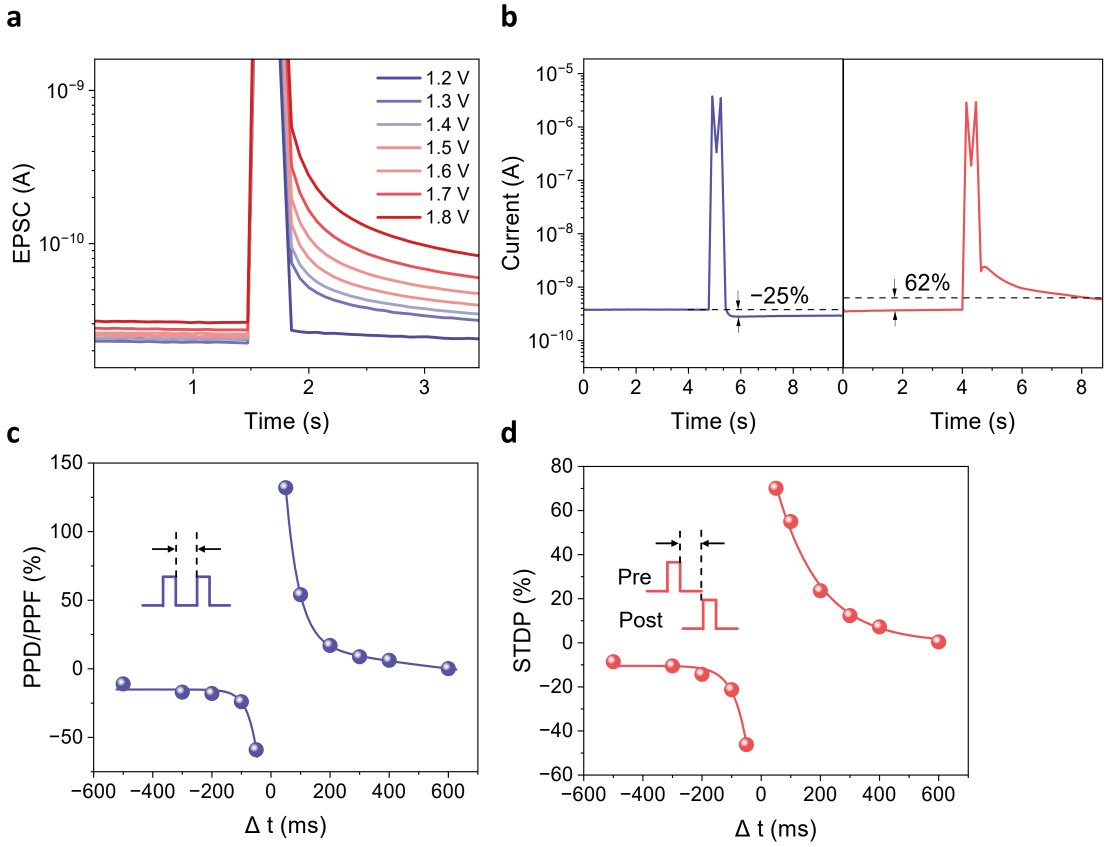

Figure S9. (a) EPSC test. Pulse width: 150 ms. (**b,c**) PPD/PPF test. Pulse amplitude/width: 1 V/150 ms. (**d**) STDP test. Pulse amplitude/width: 1V/150 ms.

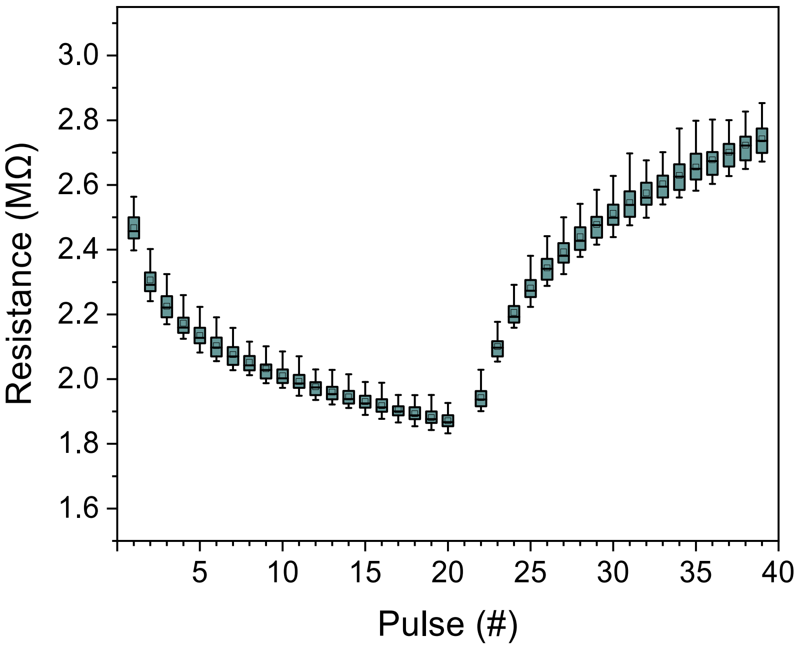

Figure S10. Box plot of pulse number dependence of memristor resistance for 60 cycles. Potentiation/depression voltage: ±0.7 V/10 ms.

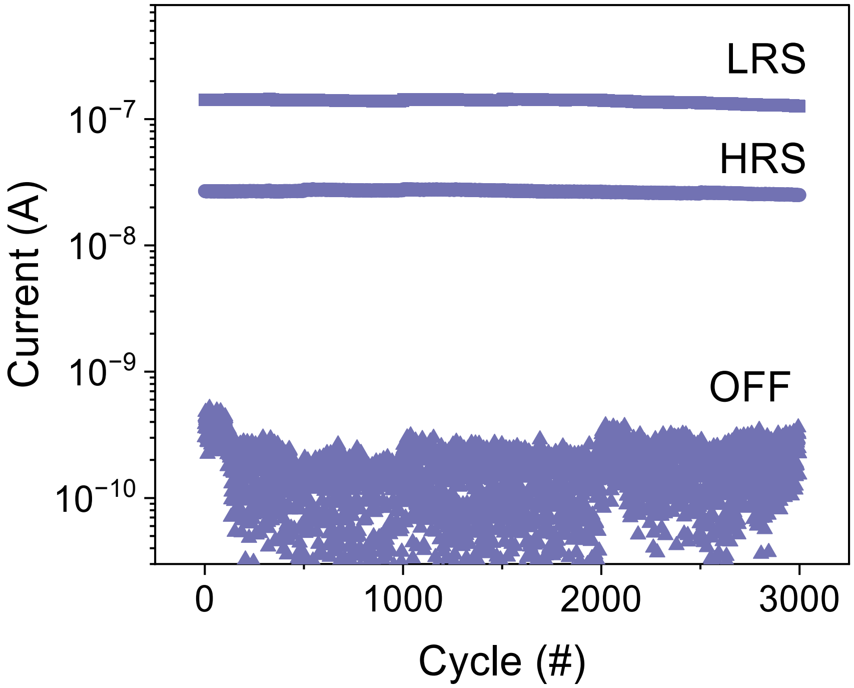

Figure S11. Endurance test for both ON and OFF state. Read pulses: 1 V/1 ms. Write pulse: 3 V/1 ms.

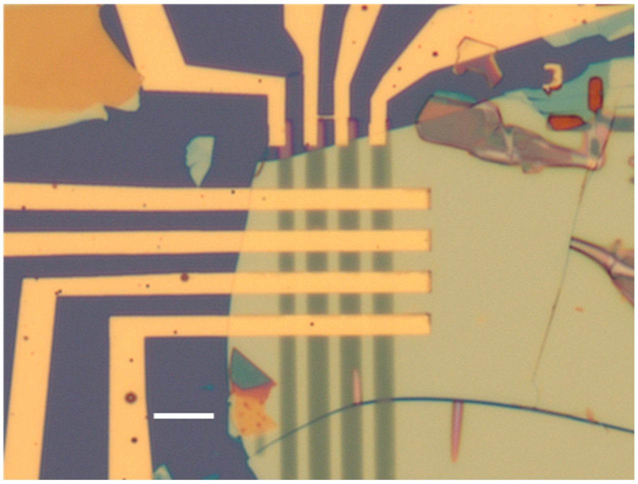

Figure S12. 1M array used for comparison to 1T1M array shown in Figure 4h. Scale bar: 10 µm. Thickness: 30 nm.

Materials	Structure	Sneak current	Path ON voltage	Path OFF voltage	Type	Tuning ratio	Ref	All vdW
MoS_2/Al_2O_3	Mem-transistor	10^{-10} A	8 V	0 V	Defect migration	10^3	1	--
MoS_2/HfO_x	1T4R	10^{-11} A	−10 V	0 V	Filament	10^6	2	--
Graphene/ WS_2/ HfO_x	1S1R	10^{-6} A	−1.5 V	−1 V	Filament	10^2	3	--
MoS_2/Al_2O_3	1T1M	10^{-11} A	8 V	−6 V	Charge trapping	10^5	4	--
Graphene/ *h*-BN/WSe_2	1D1S	10^{-10} A	6 V	3 V	Tunneling	10^5	5	✓
**This work**	1T1M	10^{-13} A	−0.5 V	−2 V	FE	10^6	--	✓

Table S1. List of representative 2D-material-based device structures for solving sneak path issues

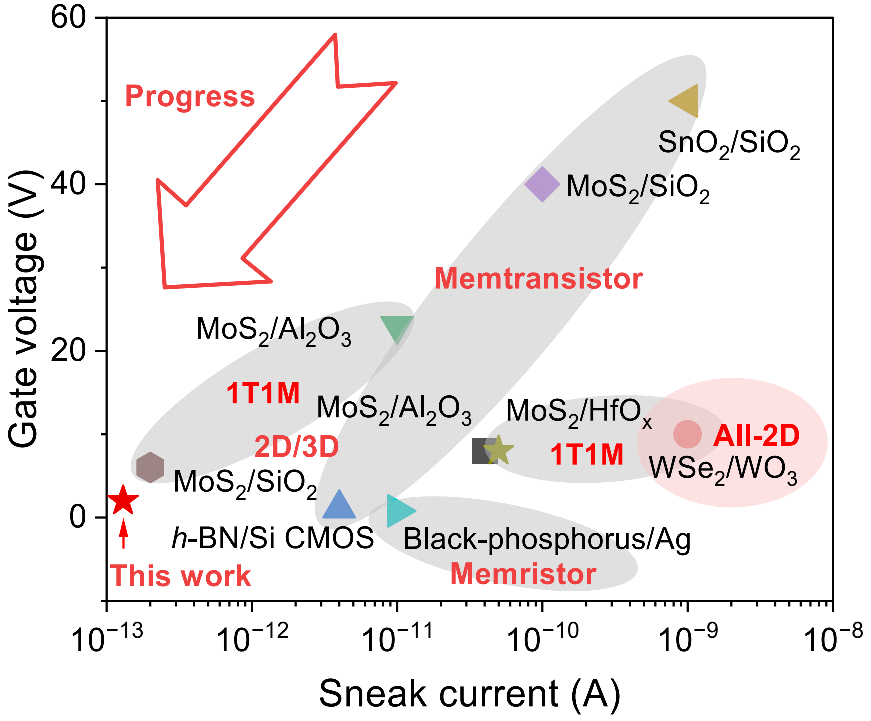

Figure S13. Comparison of various 2D-material-based devices for addressing sneak path issues[1,2,4,6-11].

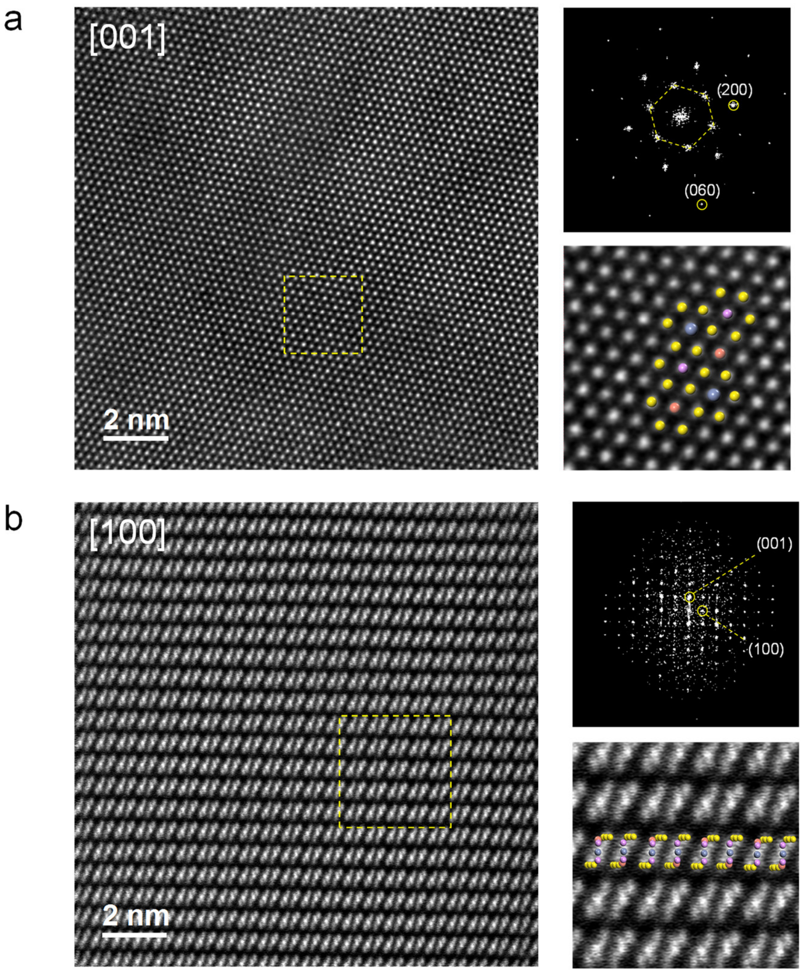

Figure S14. High-resolution transmission electron microscopy images with selected area electron diffraction patterns of $CuCrP_2S_6$ along (a) [001] and (b) [100] showing signal crystal structure with no defects, vacancies, or grain boundaries.

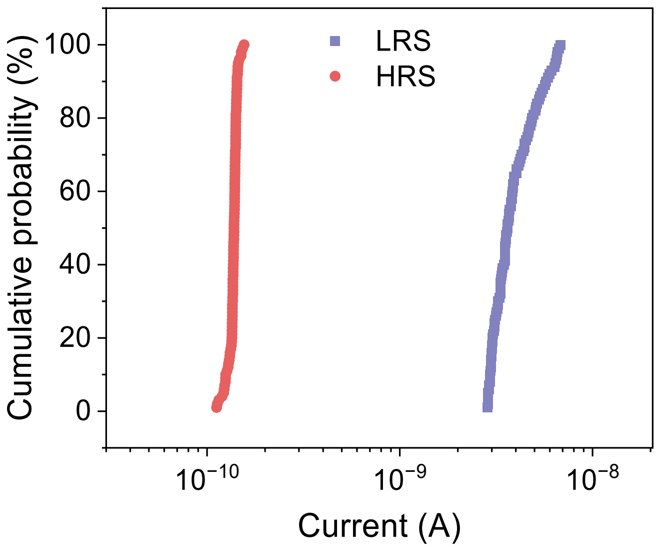

Figure S15. Cumulative distribution plot of the HRS and LRS current obtained from the 100 fully *I*–*V* sweeping cycles, extracted from Figure 2i. The relative standard deviation of the LRS and HRS over 100 cycles was calculated to be 22.16% and 5.35%, respectively.

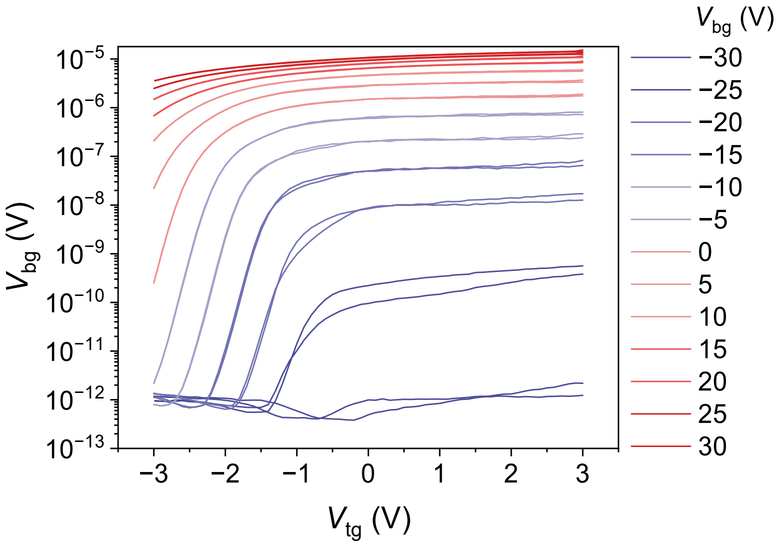

Figure S16. LRS current under various V_{tg} and V_{bg}.

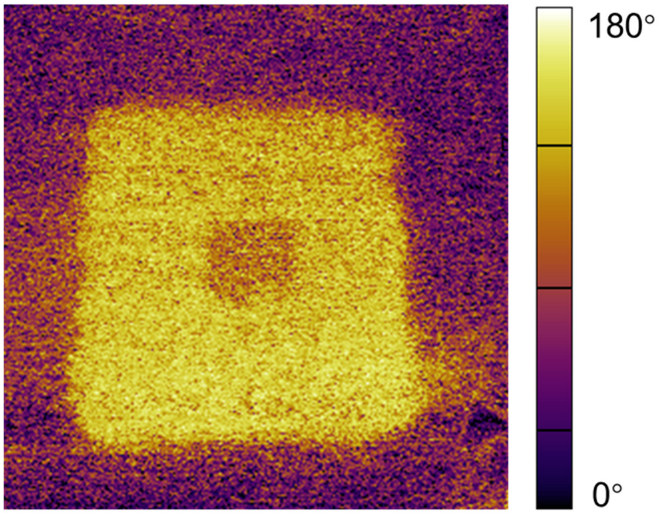

Figure S17. PFM phase image showing high contrasts of written box-in-box pattern. Frame size: 6 μm.

Experimental Methods

Material Growth and Characterization

The chemical vapor transport method was employed to synthesize $CuCrP_2S_6$ single crystals. The process involved preparing a stoichiometric mixture of Cu, Cr, P, and S with proportions of 1:1:2:6 and a total mass of 1 g. Additionally, 80 mg of iodine was added as a transport agent into the precursor mixture. This mixture was sealed in a quartz ampule within an argon-filled glove box. The ampule was connected to a mechanical pump, pumped down to 10^{-4} mbar, and resealed. The ampule was placed in a two-zone temperature furnace, with the hot and cold ends being set at 750 and 700 °C, respectively, which corresponds to a temperature gradient of 3 °C/cm. The heat treatment endured for a week, after which it was cooled down to room temperature at a gradual rate of 0.7 °C /min. A Bruker D8 Advance X-ray diffractometer employing Cu Kα radiation was used to collect the XRD pattern. We performed the Raman measurement using a WITec alpha300 apyron confocal Raman microscope with a 532 nm excitation laser, whose power was kept below 0.5 mW.

Transmission Electron Microscopy Imaging

The $CuCrP_2S_6$ and MoS_2/*h*-BN lamellar samples for transmission electron microscopy imaging were prepared using the focused ion beam milling technique on an FEI Helios G4 UX FIB-SEM system. The thickness of the lamellae was around 70 nm. The lamellar was inspected using FEI Titan Themes Cubed G2 300 (Cs Probe) TEM.

Device Fabrication

The single-crystal *h*-BN, MoS_2, and $CuCrP_2S_6$ were exfoliated on SiO_2 (300 nm)/Si wafer and then stacked with standard dry transfer technique using a PDMS polymer stamp. Metal electrodes (Ti 10 nm/Au 60 nm) were patterned by electron beam lithography (model: CABL-9000C), followed by metal sputtering and lift-off processes.

Ferroelectric Characterization

PFM measurements were carried out using an Asylum Research MFP-3D scanning probe microscope in dual-AC resonance tracking PFM (DART-PFM) mode on conductive gold-coated substrates. A Pt/Ir-coated conductive tip (with a spring constant of 3 N/m, procured from Bruker) was used. The PFM mapping was obtained with an applied AC bias of 0.8 V on the probe. The SHG measurements were carried out using an MStarter 100 Ultrafast SHG microscope spectrometer (sourced from Nanjing Metatest Optoelectronics Corporation) equipped with an excitation light source of a 1064 nm picosecond pulse laser.

Device Characterization

All electrical measurements for memristor were conducted utilizing a Keithley 4200 semiconductor parameter analyzer. The *I*–*V* curves were collected at a quiet sweep mode of Keithley 4200. All measurements were conducted in air at room temperature in the dark chamber to preclude potential interference from the photoelectric effect.

DFT Simulation

The Vienna ab initio simulation package (VASP) was employed to perform first-principles calculations. We used the projector-augmented wave method[12] and the Perdew–Burke–Ernzerhof exchange-correlation functional[13] for the simulations. A plane-wave kinetic energy cut-off of 450 eV was chosen. For structural relaxations, a Gamma-centered k-point grid with

ka > 17 Å was used to sample the Brillouin zone, where k represents the number of mesh points and a represents the lattice parameters of the supercell. A similar k-point mesh was used– for all static runs of electronic optimization. To prevent any spurious interaction between periodically repeated layers, a large vacuum space of 20 Å in the direction of c was applied. All structures were fully relaxed until the energy converged within 10^{-6} eV, and the forces converged within 5×10^{-3} eV/Å. To describe vdW forces, the DFT-D3 method with Becke–Johnson damping functions was used[14,15].

SPICE Simulations

The single layer perceptron neural networks were simulated in H-SPICE, considering all the active devices (transistors) from a 180 nm commercially available CMOS process. The synaptic weights were obtained by *ex-situ* training performed in Python using the Tensorflow and Keras libraries with the Adam optimizer and a learning rate of 0.01, achieving 90% accuracy in all cases. Each resulting synaptic weight (W_M) was then mapped to a pair of memristor devices (for which G_{HRS} = 0.1 nS and G_{LRS} = 10 nS), as indicated by Equations (1) and (2), to allow the representation of positive and negative weights, resulting in a crossbar of 18 × 6 (108 devices).

$$g_{i,j} = g_{i,j}^{+} - g_{i,j}^{-} \tag{1}$$

$$G_M^{+,-} = \frac{G_{max} - G_{min}}{max\{W_M\} - min\{W_M\}} w_M^{+,-} + \left[G_{max} - \frac{(G_{max} - G_{min}) max\{W_M\}}{max\{W_M\} - min\{W_M\}} \right] \tag{2}$$

The sub-matrix of positive and negative synaptic weights is obtained by applying Equations (3) and (4).

$$w_{M_{i,j}}^{+} \begin{cases} w_{M_{i,j}}, & w_{M_{i,j}} > 0 \\ 0, & w_{M_{i,j}} \leq 0 \end{cases} \tag{3}$$

$$w_{M_{i,j}}^{-} \begin{cases} 0, & w_{M_{i,j}} \geq 0 \\ -w_{M_{i,j}}, & w_{M_{i,j}} < 0 \end{cases} \tag{4}$$

Memristors are modelled with the Dynamic Memdiode Model (DMM) model of the memristor[16,17]. The DMM model is a behavioral model in which the electron flow is controlled by some kind of potential barrier, whose details are not specified within the framework of the model. It is based on two equations: one for the electron transport, expressed as a diode with series resistance, and another for the memory state of the device, which is based on a hysteresis operator. The resistance between adjacent memristors (line resistance) in the crossbar varied from 1 to 1000 Ω, revealing a clear impact. The input signals fed to the neural networks were obtained by rearranging into an $n^2 \times 1$ format the pixels of the train/test images from MNIST datasets, which were previously rescaled to different n × n sizes (8 × 8, 12 × 12, and 16 × 16 pixels) using the bipolar interpolation algorithm. Subsequently, the intensity of each pixel was translated into an analog voltage ranging from 0 to 200 mV. The signals propagated from each output neuron were post-processed in Python to determine the neuron with the highest firing

rate during each time frame ("Soft-Arg-max" fashion), thereby determining the predicted pattern. Further details regarding the implemented procedure can be found in Refs[17-19].

References

1. Feng, X., Li, S., Wong, S. L., Tong, S., Chen, L., Zhang, P., Wang, L., Fong, X., Chi, D., Ang, K. W. Self-selective multi-terminal memtransistor crossbar array for in-memory computing. *ACS Nano* **15**, 1764-1774 (2021).
2. Xie, M., Jia, Y., Nie, C., Liu, Z., Tang, A., Fan, S., Liang, X., Jiang, L., He, Z., Yang, R. Monolithic 3D integration of 2D transistors and vertical RRAMs in 1T-4R structure for high-density memory. *Nat. Commun.* **14**, 5952 (2023).
3. Shen, M., Shen, S., Jia, Y., Liu, Y., Zhang, P., Xie, M., Wei, J., Yang, R. One-selector-one-resistor integrated memory cells based on two-dimensional heterojunction memory selectors. *ACS Nano* **18**, 28292-28300 (2024).
4. Fu, S., Park, J. H., Gao, H., Zhang, T., Ji, X., Fu, T., Sun, L., Kong, J., Yao, J. Two-terminal MoS_2 memristor and the homogeneous integration with a MoS_2 transistor for neural networks. *Nano Lett.* **23**, 5869-5876 (2023).
5. Wang, X., Qiao, R., Lu, H., He, W., Liu, Y., Zhou, T., Wan, D., Wang, Q., Liu, Y., Guo, W. 2D memory selectors with giant nonlinearity enabled by van der Waals heterostructures. *Small* **20**, e2310158 (2024).
6. Ding, G., Yang, B., Chen, R. S., Mo, W. A., Zhou, K., Liu, Y., Shang, G., Zhai, Y., Han, S. T., Zhou, Y. Reconfigurable 2D WSe_2-based memtransistor for mimicking homosynaptic and heterosynaptic plasticity. *Small* **17**, e2103175 (2021).
7. Huang, C. H., Chang, H., Yang, T. Y., Wang, Y. C., Chueh, Y. L., Nomura, K. Artificial synapse based on a 2D-SnO_2 memtransistor with dynamically tunable analog switching for neuromorphic computing. *ACS Appl. Mater. Interfaces* **13**, 52822-52832 (2021).
8. Zhu, K., Pazos, S., Aguirre, F., Shen, Y., Yuan, Y., Zheng, W., Alharbi, O., Villena, M. A., Fang, B., Li, X., Milozzi, A., Farronato, M., Munoz-Rojo, M., Wang, T., Li, R., Fariborzi, H., Roldan, J. B., Benstetter, G., Zhang, X., Alshareef, H. N., Grasser, T., Wu, H., Ielmini, D., Lanza, M. Hybrid 2D-CMOS microchips for memristive applications. *Nature* **618**, 57-62 (2023).
9. Sangwan, V. K., Lee, H. S., Bergeron, H., Balla, I., Beck, M. E., Chen, K. S., Hersam, M. C. Multi-terminal memtransistors from polycrystalline monolayer molybdenum disulfide. *Nature* **554**, 500-504 (2018).
10. Ahmed, T., Kuriakose, S., Tawfik, S. A., Mayes, E. L. H., Mazumder, A., Balendhran,

S., Spencer, M. J. S., Akinwande, D., Bhaskaran, M., Sriram, S., Walia, S. Mixed ionic-electronic charge transport in layered black-phosphorus for low-power memory. *Adv. Funct. Mater.* **32**, 2107068 (2021).

11. Wang, C. H., McClellan, C., Shi, Y., Zheng, X., Chen, V., Lanza, M., Pop, E. and Wong, H.S.P. 3D monolithic stacked 1T1R cells using monolayer MoS2 FET and hBN RRAM fabricated at low (150°C) temperature. In: *IEEE International Electron Devices Meeting (IEDM)*). IEEE (2018).
12. Kresse, G. From ultrasoft pseudopotentials to the projector augmented-wave method. *Phys. Rev. B.* **59**, 1758 (1999).
13. Perdew, J. P., Burke, K., Ernzerhof, M. Generalized gradient approximation made simple. *Phys. Rev. Lett.* **77**, 3865 (1996).
14. Grimme, S., Antony, J., Ehrlich, S., Krieg, H. A consistent and accurate ab initio parametrization of density functional dispersion correction (DFT-D) for the 94 elements H-Pu. *J. Chem. Phys.* **132**, 154104 (2010).
15. Grimme, S., Ehrlich, S., Goerigk, L. Effect of the damping function in dispersion corrected density functional theory. *J. Comput. Chem.* **32**, 1456-1465 (2011).
16. Aguirre, F. L., Sune, J., Miranda, E. SPICE implementation of the dynamic memdiode model for bipolar resistive switching devices. *Micromachines* **13**, 330 (2022).
17. Aguirre, F. L., Pazos, S. M., Palumbo, F., Suñé, J., Miranda, E. SPICE simulation of RRAM-based cross-point arrays using the dynamic memdiode model. *Front. Phys.* **9**, 735021 (2021).
18. Aguirre, F. L., Pazos, S. M., Palumbo, F., Sune, J., Miranda, E. Application of the quasi-static memdiode model in cross-point arrays for large dataset pattern recognition. *IEEE Access* **8**, 202174-202193 (2020).
19. Aguirre, F. L., Gomez, N. M., Pazos, S. M., Palumbo, F., Suñé, J., Miranda, E. Minimization of the line resistance impact on memdiode-based simulations of multilayer perceptron arrays applied to pattern recognition. *J. Low Power Electron. Appl.* **11**, 9 (2021).
